# Oral-Health-Related Quality of Life in Adult Patients with Rheumatic Diseases—A Systematic Review

**DOI:** 10.3390/jcm9041172

**Published:** 2020-04-19

**Authors:** Gerhard Schmalz, Susann Patschan, Daniel Patschan, Dirk Ziebolz

**Affiliations:** 1 Department of Cariology, Endodontology and Periodontology, University of Leipzig, 04103 Leipzig, Germany; dirk.ziebolz@medizin.uni-leipzig.de; 2 Department of Cardiology, Angiology and Nephrology, Klinikum Brandenburg, Medizinische Hochschule Brandenburg, 14770 Brandenburg, Germany; spatschan@gmail.com (S.P.); d.patschan@gmail.com (D.P.)

**Keywords:** rheumatoid arthritis, oral health-related quality of life, quality of life, systemic sclerosis, Sjörgen syndrome

## Abstract

Objectives: The aim of this systematic review was to assess the oral-health-related quality of life (OHRQoL) of adult patients with rheumatic diseases. Material and Methods: A systematic literature search was performed, including clinical studies on adults (aged at least 18 years) with a verified diagnosis of rheumatic disease. Results: 26 out of 41 clinical studies including rheumatoid arthritis (RA, seven studies), systemic sclerosis (SSc, five), Sjögren syndrome (SS, eight), Behcet disease (BD, four), systemic lupus erythematosus (SLE, one) and ankylosing spondylitis (AS, one) were found. In 15 studies, a healthy control group was recruited. The short form of the Oral Health Impact Profile (OHIP 14) was most frequently applied. The majority of studies (14/15) reported worse OHRQoL in patients with rheumatic disease compared to healthy individuals. In particular, patients with SS (salivary flow and composition) or BD (oral ulcers) showed a relation between OHRQoL and disease-specific oral manifestations. Most studies investigating subscales of OHRQoL (5/6) found the subscale physical disability to be predominantly affected in patients with rheumatic diseases. About half of the studies reported impaired psychosocial aspects. Conclusion: Patients with rheumatic diseases exhibit reduced OHRQoL, especially in diseases with oral manifestations like SS and BD. Physical affections due to oral diseases and psychosocial impairments caused by disease-related parameters must be recognized within patient-centered dental care.

## 1. Introduction

Autoimmune rheumatic diseases are a heterogeneous group of disorders causing high morbidity in affected patients. Therefore, regardless of their rare occurrence, the clinical relevance of these diseases appears to be high [1]. While the progress in therapeutic management of rheumatic disorders has been enormous in recent decades, one focus in the care of these patients is their quality of life [2]. While quality of life is a broad area, the oral-health-related quality of life (OHRQoL) represents a partial aspect of the general health-related quality of life (HRQoL) [3]. This OHRQoL reflects the individual, subjective perception of the oral health status of a patient, and is primarily related to the physical oral conditions, but also to general HRQoL issues [4]. Thereby, different dimensions of OHRQoL exist, including psychosocial and functional issues, whereby many patient-reported outcome measures are available, which differ regarding their response format, number of items, context of use, or target group [5].

Oral diseases, especially periodontitis and tooth loss, are highly prevalent in patients with rheumatic diseases [6]. This could be related to patients’ OHRQoL. Thereby, oral manifestations of rheumatic diseases like reduced salivary flow in patients suffering from Sjögren syndrome or oral ulcers in Behcet’s diseases, periodontal manifestations in rheumatoid arthritis, or side-effects of anti-rheumatic drugs might be of relevance [6]. The administration of some biological drugs in the treatment of rheumatic diseases could be related to medication-related osteonecrosis of the jaw (MRONJ), such as that associated with the use of tocilizumab in the treatment of rheumatoid arthritis [7]. Alongside with this clinical impact of rheumatic disease on oral health, the reduced general HRQoL related to rheumatic diseases [2] might be another influential factor on patients’ OHRQoL. Accordingly, the OHRQoL of patients suffering from rheumatic diseases and its potential associations with oral health as well as disease-specific parameters are an issue of interest. While several studies were able to show reduced OHRQoL in patients with rheumatic diseases, the results were heterogeneous and different patient groups, inclusion criteria, clinical procedures, and OHRQoL measurement instruments were applied [8,9,10,11,12,13,14,15,16,17]. Thus, the application of different dimensions or subscales of OHRQoL in patients with rheumatic diseases remains particularly unclear. Furthermore, the validity of the applied OHRQoL measurements can be discussed. 

Therefore, this systematic review following Preferred Reporting Items for Systematic Reviews and Meta-Analyses (PRISMA) guidelines [18] aimed at the comprehensive assessment of clinical studies investigating the OHRQoL of adult patients with rheumatic diseases. The focus was therefore set to cover three major issues: (I) the OHRQoL between different rheumatic diseases and its differences from healthy controls, as well as its relationship to general HRQoL, oral health, and disease-specific parameters; (II) the application of different subscales of OHRQoL in patients with rheumatic disease; and (III) the applied OHRQoL measurements with respect to their validity in the patient group. With this information, conclusions were made regarding the relationship between physical oral health, and also disease-related, HRQoL-related, and psychosocial factors with the OHRQoL of patients suffering from rheumatic diseases.

## 2. Methods

The authors followed the criteria established in the Preferred Reporting Items for Systematic Reviews and Meta-Analyses (PRISMA) guidelines for this review [18].

### 2.1. PICO (Patient Intervention Control Outcome) Question

Based on the heterogeneity of the available data, the existing literature regarding OHRQoL of patients with different systemic rheumatic diseases was reviewed. The following primary question was formed: Is there a difference between different entities of systemic rheumatic diseases and healthy controls?

### 2.2. Search Strategy

A systematic search was performed by two independent reviewers based on the PubMed, Scopus, and Cochrane databases in January 2020 using the following search terms: “rheumatic diseases” OR “rheumatoid arthritis” OR “lupus” OR “systemic sclerosis” OR “Sjögren syndrome” OR “ankylosing spondylitis” OR “vasculitis” OR “Behcet’s disease” AND "oral-health-related quality of life." Based on the findings, an additional manual search considering the references of the included studies was executed.

### 2.3. Inclusion and Exclusion Criteria

The search results were screened and checked for eligibility with regard to previously formed criteria. Only full-text articles in English that were published after the year 1999 were considered. Mandatory condition for inclusion included the recruitment of patients suffering from a systemic rheumatic disease with a verified diagnosis by clearly defined, disease-specific criteria within a clinical study. Furthermore, only studies explicitly reporting on any OHRQoL outcome were included. Only adult patients were examined. Therefore, studies with patients <18 years of age were excluded. 

### 2.4. Selection of the Studies

Following the PRISMA guidelines [18], duplicate findings were checked and removed as a first step. Second, abstracts of the findings from the systematic search were screened. After screening, full-text articles were carefully reviewed regarding the in- and exclusion criteria, and included in the further data extraction procedure if they fulfilled these criteria. 

### 2.5. Data Extraction

For the qualitative analysis, the following major information was extracted from the included investigations:

- Form of disease, year of publication, number of participants, study setting, age, sex, and disease duration of the diseased group;
- Recruitment of a healthy control group for comparison of OHRQoL findings;
- Form of OHRQoL assessment, results of OHRQoL and its potential relationship to general HRQoL, oral-health- and/or disease-specific parameters;
- If applicable, results for subscales of the OHRQoL measurements and whether they were worse than for healthy control individuals;
- If applicable, information regarding the validity of the applied measurements.

All clinical studies were screened for this information. If studies included patients who were part of previously published investigations, it was checked whether there were repetitious results. Only if other in- and exclusion criteria, other examination procedures, other OHRQoL assessment measures, or additional information was available were both studies included. The whole systematic review process, including literature search, abstract/title/full-text screening, and extraction of data, was accomplished by two independent individuals. 

## 3. Results

### 3.1. Search Findings

**Figure 1:**
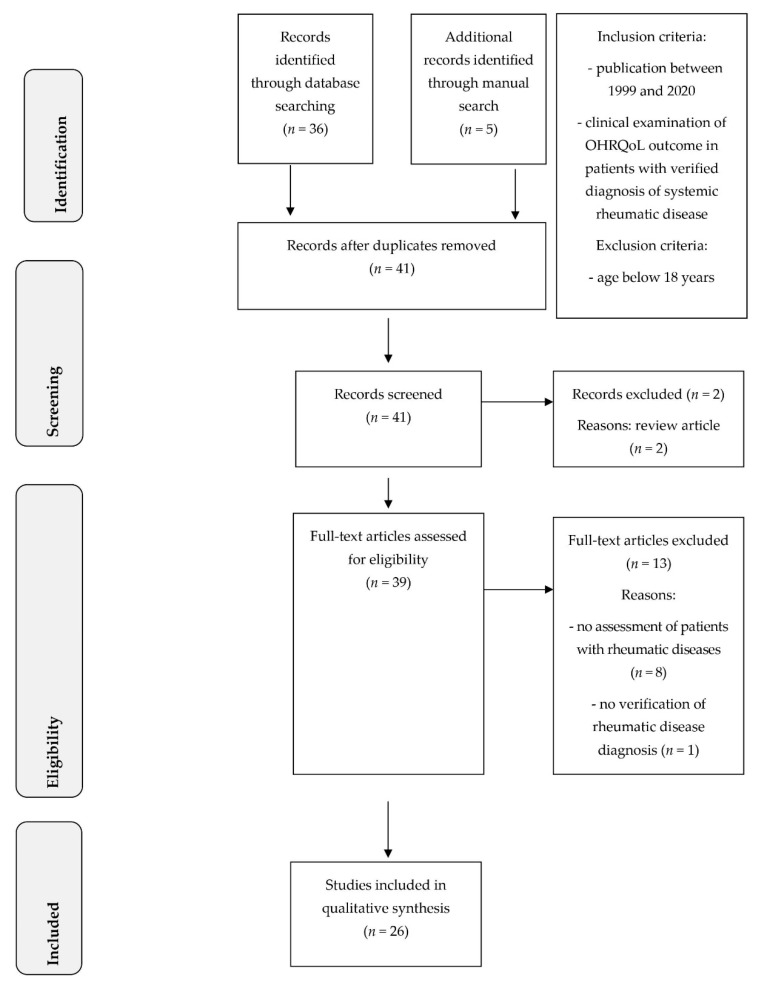
PRISMA (Preferred Reporting Items for Systematic Reviews and Meta-Analyses) diagram for systematic review process.

The systematic search revealed 41 articles, which were found by the applied search terms complemented by manual search. Two articles were reviews and thus were excluded from further process. A total of 39 full-text articles were screened with regard to the defined in- and exclusion criteria. Accordingly, 13 articles were excluded for the following reasons: one article did not assess OHRQoL in rheumatic diseases at all; one questionnaire-based examination did not verify the diagnosis of rheumatic disease; and seven studies were performed in patients with temporomandibular disorders, including craniomandibular dysfunction or osteoarthritis restricted to the temporomandibular joint. Furthermore, four studies included patients with juvenile idiopathic arthritis and were excluded because of their inclusion of patients with an age below 18 years (Supplementary Table S1). Consequently, a total of 26 eligible studies were included in the qualitative analysis of this systematic review (Figure 1).

### 3.2. Characteristics of Included Studies

The following rheumatic diseases were subject of the included investigations: rheumatoid arthritis (RA, seven studies), systemic sclerosis (SSc, five studies), Sjögren syndrome (SS, eight studies), Behcet’s disease (BD, four studies), systemic lupus erythematosus (SLE, one study), and ankylosing spondylitis (AS, one study). Regarding study type, the majority of examinations were cross-sectional studies, wherein 4 were multi-centric and 18 monocentric. Two studies were randomized clinical trials and two investigations were observational with a follow-up of two weeks or three years, respectively. The number of included patients differed between 20 and 675 participants with rheumatic diseases. In 17 studies, a control group was recruited (15 healthy controls, 2 other controls). The main study characteristics are presented in Table 1. 

### 3.3. OHRQoL Measurements and Results

**Figure 2:**
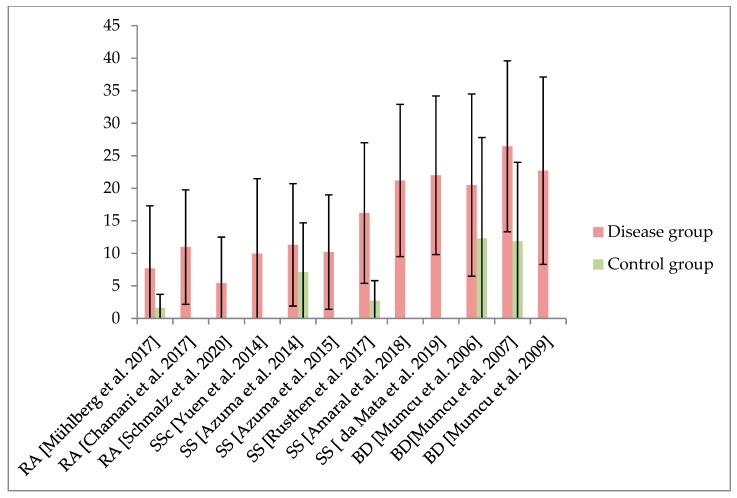
Findings of OHIP (oral health impact profile) 14 in patients suffering from rheumatic diseases, including rheumatoid arthritis (RA), systemic sclerosis (SSc), Sjögren syndrome (SS), and Behcet’s disease (BD). For comparability, only studies presenting mean value ± standard deviation are displayed in this figure. If applicable, results of the control group are presented as well.

**Figure 3:**
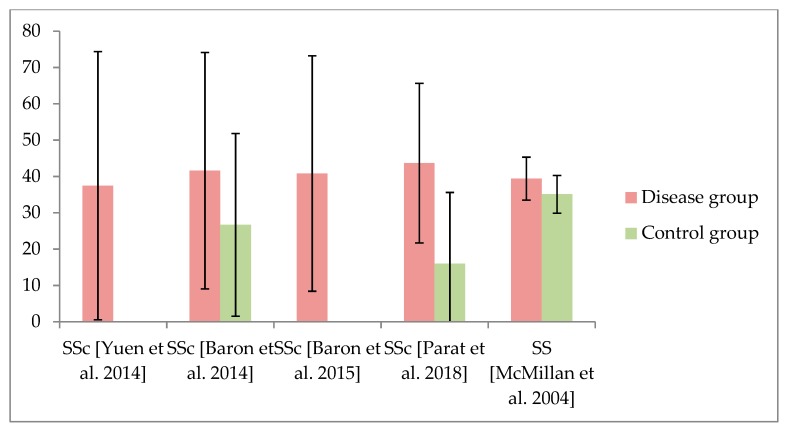
Findings of OHIP 49 in patients suffering from rheumatic diseases, including systemic sclerosis (SSc) and Sjögren syndrome (SS). For comparability, only studies presenting mean value ± standard deviation are displayed in this figure. If applicable, results of the control group are presented as well.3.4. OHRQoL Subscales

The main findings of the included studies are presented in Table 2. The measurement reported most often was the short form of the Oral Health Impact Profile (OHIP 14), which was applied in 15 of the included studies. The extended version, including 49 questions (OHIP 49), was reported in seven studies. Used in only four studies, the General Oral Health Assessment Index (GOHAI) was rarely applied. The Quality of Life in Xerostomia Questionnaire (XeQoL), the Mouth Handicap in Systemic Sclerosis (MHISS) assessment, or a specifically composed OHRQoL questionnaire were used only once each. The results of OHIP 14 and OHIP 49 are presented in Figure 2 and Figure 3 and Table 2. Of the 15 investigations that compared the OHRQoL of a patient group suffering from rheumatic disease with healthy controls, 14 studies reported worse OHRQoL in rheumatic-diseased individuals. The relationship between OHRQoL and general HRQoL has rarely been examined. Results regarding potential associations and/or correlations between OHRQoL and oral health, as well as rheumatic-disease-specific parameters, were heterogeneous (Supplementary Table S2). Patients with Sjögren syndrome (salivary flow and composition) or Behcet's disease (oral ulcers) showed a particular relationship between OHRQoL and disease-specific oral manifestations (Table 2). 

A total of eleven studies reported subscales of the OHRQoL measurement, of which five reported OHIP 14 subscales, five reported OHIP 49 subscales, and one study examined the subscales of XeQoL. Within six studies, the subscales were compared between rheumatic-diseased individuals and a healthy control group. The majority of these studies (5/6) found the OHRQoL associated with physical disability to be lower in rheumatic-diseased patients. About half of the studies reported impaired psychosocial aspects (Table 3 and Table 4). The study that applied the XeQoL found significantly worse results for the subscales related to physical pain, psychosocial discomfort, physical disability, and social disability in individuals with SS compared to healthy controls [8].

### 3.4. Validity of Applied OHRQoL Measurement 

Seven of the included investigations provided information about the validity of the applied measurement. The intraclass correlation coefficient fell between 0.61 to 0.94, and the Cronbach’s α was determined to be between 0.89 and 0.99 (Table 5). However, one study that explicitly focused on the reliability of OHIP 49 in SSc reported a large standard error of measurement and concluded OHIP 49 to be not precise and sensitive for these patients [19].

## 4. Discussion

This is the first systematic review of OHRQoL in patients with different rheumatic diseases. The qualitative analysis revealed 26 clinical studies, from which six disorders, i.e., RA, SSc, SS, SLE, AS, and BD, were included in the review. In accordance to the World Dental Federation (FDI) definition of oral health as a synthesis of physical and psychological well-being with regard to the orofacial system [35], the OHRQoL plays an indispensable role in dental care. The concept of OHRQoL enables a shift from the pure physical consideration of the patients with a focus restricted to the haptic presence of oral diseases to a comprehensive patient-centered assessment [4,36]. Thereby, OHRQoL must be seen as a mandatory sub-aspect of general HRQoL, including the possible interrelationship between orofacial health and general HRQoL in a complex interaction [3,4,36]. Accordingly, the assessment of OHRQoL in the context of oral-health- and disease-related parameters in patients with rheumatic diseases appears to be contemporary and reasonable. However, rheumatic diseases constitute a heterogeneous group of different disorders with manifold therapeutic strategies [1], making comparison and interpretation difficult. Moreover, the inconsistency of included studies regarding their study design, patient selection criteria, methodology, and geographical specifics has an impact on their comparability. Nevertheless, the results of this systematic review can provide some interesting information for the interdisciplinary dental care of patients with rheumatic diseases. Moreover, recommendations for future research in the field can be formulated. 

Generally a reduced OHRQoL is apparent in patients with rheumatic disease. A total of 15 studies reported the OHRQoL in comparison with a healthy control group, of which 14 studies found worse results in patients suffering from rheumatic diseases [8,11,12,13,15,16,21,22,24,26,30,31,33,34]. The valid interpretation of further results is difficult. Reference values for generally healthy individuals can help to provide a statement as to whether results represent a deficit in the OHRQoL of the patients. Due to cultural differences as well as a large influence of age, sex, and the presence of teeth/prosthodontic treatment on OHRQoL outcome [37,38], consistent international reference values are not available. For instance, the German dentate general population exhibits a sum score of OHIP 14 of 0–4 or OHIP 49 between 5 and 15 points depending on full or partial dentition [38,39]. The comparability is limited due to different countries with particularities potentially affecting OHRQoL [37]. Irrespective of this fact, the OHIP values of all included studies with patients suffering from rheumatic diseases were higher than these reference values. Within the studies that applied the OHIP questionnaire to patients with rheumatic diseases, SS and BD in particular showed the highest OHIP scores, representing a reduced OHRQoL (Figure 2 and Figure 3). SS is a chronic inflammatory disorder primarily affecting the exocrine glands, what leads to a dryness of mouth and eyes due to involvement of salivary and lacrimal glands, respectively [40]. The resulting xerostomia is a complex condition, leading to remarkable impairment of OHRQoL [41]. Accordingly, majority of included studies dealing with SS showed associations between worse OHRQoL and salivary flow or composition [8,24,25,26,28]. One study that examined RA also investigated the influence of xerostomia on OHRQoL and was also able to show a negative impact [14]. The disease-related xerostomia in patients with SS might therefore be a major reason for their impaired OHRQoL. Similarly, BD represents a chronic multisystem disease with a special disease-related condition affecting the oral cavity: oral ulcers [42]. This painful efflorescence of oral soft tissues constitutes a logical reason for their impaired OHRQoL. This was confirmed by the negative effect of active ulcers on OHRQoL in the included studies [30,31,32,33]. Therefore, rheumatic diseases leading to a primarily oral manifestation appear to have the largest impact on OHRQoL among the included investigations. In this context, the relationship between periodontaitis and rheumatic diseases, especially RA, needs to be mentioned [43]. Although RA is related to a higher prevalence and increased severity of periodontitis [6], this appears to have no remarkable effect on patients’ OHRQoL, because none of the included studies reported associations between periodontitis and OHRQoL explicitly (Table 2). Within the oral conditions, only the presence of missing/extracted teeth and/or wearing dentures was repeatedly reported to affect OHRQoL [9,14,17,30,32,34]. It appears evident that tooth loss can be seen as a factor influential on OHRQoL, independently of the applied instrument for measurement or included patients [44]. Accordingly, this finding appears not to be specific for rheumatic diseases. However, the higher risk for tooth loss in rheumatic diseases, especially RA, due to the relationship to periodontal burden should be recognized in this context [45]. Considering both the influence of tooth loss on OHRQoL in general [44] and the high prevalence of tooth loss in rheumatic disease [6,45], this is an important issue. On one hand, the avoidance of tooth loss might be of increasing relevance for these patients to reduce the impairment of their OHRQoL. On the other hand, tooth loss or denture wearing should be assessed and considered in studies dealing with the OHRQoL of patients suffering from rheumatic diseases. 

Another issue touched on by this systematic review is the occurrence of associations between general HRQoL and/or rheumatic-disease-related parameters with OHRQoL. As presented in Table 2, results regarding this issue are heterogeneous and only a small number of studies also examined HRQoL. For generally healthy individuals, a relationship between OHRQoL and general HRQoL is documented in literature, whereby OHRQoL is more strongly related to physical oral health than to general HRQoL [4,36]. However, patients with rheumatic diseases often show reduced HRQoL due to their disease burden, pain, anxiety, or depression [2]. Therefore, the interrelation between OHRQoL and general HRQoL is of interest in these patient groups. The majority of included studies (5/6) that examined this relationship found associations, whereby different measurements for HRQoL were applied [8,9,15,21,22]. Two potential explanations might be conceivable for this finding. On one hand, the oral conditions related to rheumatic diseases might have an influence on both OHRQoL and general HRQoL in these patients. Based on the literature, this seems conceivable [4,36], but because none of the studies explicitly examined this issue, this remains speculative. The second potential explanation could be the effect of rheumatic disease on whole HRQoL, including OHRQoL, due to the physical and psychosocial impairment of patients caused by the underlying disease. Accordingly, the impaired OHRQoL of these patients would be more strongly affected by rheumatic disease burden than by their oral conditions. To support this hypothesis, the associations between OHRQoL and rheumatic-disease-related parameters were examined. The majority of studies that examined this issue (16/21) were able to confirm a relationship between OHRQoL and disease-related parameters [8,12,13,14,16,17,22,24,25,26,28,30,31,32,33,34]. These parameters were based on the heterogeneity of different diverse rheumatic diseases, including age, disease duration, disease activity, or disease severity. Thus, disease-specific oral manifestations in SS (xerostomia) and BD (oral ulcers) that primarily affect oral cavity were of particular relevance, as described above [8,24,25,26,28,30,31,32,33]. The general HRQoL of patients with rheumatic diseases is affected by disease-related parameters like age, disease duration, and disease severity [46,47]. The relationship between OHRQoL and disease-related parameters as assessed in the majority of studies might thus imply that this is similar for OHRQoL and for general HRQoL in these patients. 

For a deeper understanding, the subscales of OHRQoL can be considered additionally. Because five studies did not compare subscales to a healthy control [14,17,28,29,32], and different subscales were applied, the comparability is limited. However, some interesting information can be derived from the findings: In the vast majority of studies with a control group (5/6), a significant effect of the subscale “physical disability” was confirmed without any predominant manifestation in one of the included diseases [8,11,20,30,34]. Furthermore, half of examinations (3/6) also confirmed a significant impairment of the psychosocial and/or psychological sub-aspects of OHRQoL [8,20,31]. Moreover, the potential relevance of psychosocial patterns is highlighted by two special results. In a randomized clinical trial, the effect of malic acid on OHRQoL of patients with SS was tested. The largest improvement was recorded for psychological pattern [29]. Another study in patients with RA found correlations between disease activity and the pattern of psychosocial impact [17]. Rheumatic diseases constitute an important psychosocial impairment [48]. Accordingly, the relevance of psychological and/or psychosocial issues in the context of OHRQoL might be of clinical relevance. It is therefore conceivable that beside impaired physical oral health due to related manifestations like xerostomia or oral ulcers, a psychosocial impairment caused by rheumatic disease burden might be responsible for the impaired OHRQoL of these patients. This condition might be of particular relevance, because a solely physical understanding might be inadequate for their dental care. In fact, this rather argues for a patient-centered, individual, and multidisciplinary care model [49], which must also be implemented in the dental care of patients with rheumatic diseases. 

Another issue of relevance is the methodology applied in the included studies. A total of six different questionnaires, including OHIP 49, OHIP 14, GOHAI, MHISS, XeQoL, and a specific OHRQoL questionnaire, were used. The OHIP and GOHAI questionnaires appear to be valid and reliable assessment measures for OHRQoL [5]. The available examinations showed appropriate validity for the applied questionnaires, and the majority of available information was provided for OHIP questionnaire [19,28,29,30,31,32]. Only one study concluded OHIP 49 to be not precise and sensitive for patients with SSc and disease-specific questionnaires to be needed [19]. While a strong conclusion as to the most appropriate measurement tool is not possible based on the available studies, several consideration can be highlighted. The use of the OHIP questionnaire can be especially recommended for rheumatic diseases with oral manifestations because of its appropriate psychometric properties, especially in case of xerostomia [50]. This was confirmed by the results for SS and BD in the included studies. For rheumatic diseases like SSc with a lower number of oral manifestations, disease-specific questionnaires like the MHISS might be more informative and suitable [10,21]. However, for these diseases, the OHIP questionnaire, reporting mean value, standard deviation, and median are also recommended to ensure comparability with available literature. Furthermore, reference values for interpretation of OHRQoL results are recommended [38]. Therefore, future studies might focus on the evaluation of these references for rheumatic-diseased individuals. Furthermore, the clinical relevance of detected differences like OHIP scores must be considered. Interventional studies especially focus on minimal important difference as a reference for a noticeable clinical effect [51]. These values are available for systemically healthy but not rheumatic-diseased individuals, making investigations regarding this issue recommendable. Additionally, studies assessing OHRQoL of patients with rheumatic diseases should also evaluate HRQoL, oral conditions (especially missing teeth and denture wearing), and disease-related parameters to draw robust conclusions. 

Strengths and limitations: This systematic review followed the PRISMA guidelines [18,52] and was executed according to previously defined in- and exclusion criteria for the studies. Furthermore, this is the first systematic evaluation of OHRQoL in patients with different rheumatic diseases. Some limitations need to be recognized. Rheumatic diseases are heterogeneous [1], making comparison difficult. Furthermore, although in- and exclusion criteria were formulated, very different study designs with specific patient groups were included. However, the review aimed to provide a comprehensive insight of the OHRQoL in rheumatic-diseased individuals. Due to the different questionnaires and the inconsistent reporting of the results, a quantitative analysis was not possible.

## 5. Conclusions

Patients suffering from rheumatic diseases exhibit reduced OHRQoL, especially in diseases with oral manifestations like SS and BD. Physical affections due to oral diseases and psychosocial impairments caused by disease-related parameters must be recognized. Therefore, multidisciplinary, patient-centered dental care is recommendable for patients with rheumatic diseases. Future examinations should include general and disease-specific OHRQoL measurements and should apply standardized methodology and reporting of the results. The evaluation of disease-related reference values for OHRQoL and minimal important differences for the interpretation of clinical relevance should be part of future studies in the field. 

## Figures and Tables

**Table 1:** Studies included in systematic review. Values for age and disease duration are presented as mean value ± standard deviation or mean value (range).

Author, Year	Country	No of Patients	Study Setting	Subjects’ Mean Age in Years	Disease Duration	Female (%)	Control Group for OHRQoL
**Rheumatoid Arthritis**							
Blaizot et al., 2013 [9]	France	73	Monocentric cross-sectional	60.2 ± 11.9	15.2 ± 9.6 years	75.3%	no
Mühlberg et al., 2017 [13]	Germany	103	Monocentric cross-sectional	55.5 ± 11.0	11.1 ± 15.9 years	56.3%	healthy controls (*n* = 104)
Chamani et al., 2017 [14]	Iran	200	Monocentric cross-sectional	50.02 ± 13.72	62.28 ± 74.41 months	83.5%	no
Tristiu et al., 2018 [15]	Romania	91	Monocentric cross-sectional	52.82 ± 11.00	7.53 ± 7.41 years	75.8%	healthy controls (*n* = 30)
de Azevedo Branco et al., 2019 [11]	Brazil	42	Monocentric cross-sectional	52.04 ± 11.08	n/a	88.1%	healthy controls (*n* = 70)
Nosratzehi et al., 2019 [16]	Iran	80	Monocentric cross-sectional	51.6 ± 14.8	n/a	88.8%	healthy controls (*n* = 80)
Schmalz et al., 2020 [17]	Germany	176	Monocentric cross-sectional	62.5 ± 10.2	92.0 ± 102.0 months	82%	no
**Systemic Sclerosis**							
Maddali Bongi et al., 2012 [10]	Italy	40	Monocentric cross-sectional	57.27 ± 11.41	9.4 ± 4.4 years	85%	no
Yuen et al., 2014 [19]	USA	39	Single-blinded, randomized, controlled study	51.9 ± 12.8	7.8 ± 6.1 years	79.5%	no
Baron et al., 2014 [20]	Canada	163	Multicentric cross-sectional	56.20 ± 10.56	13.9 ± 8.5 years	89.6%	healthy controls (*n* = 231)
Baron et al., 2015 [21]	Canada	156	Multicentric cross-sectional	56.1 ± 10.7	13.8 ± 8.5	90.4%	non-participating subjects CSRG cohort (*n* = 1221)
Parat et al., 2018 [22]	Croatia	31	Monocentric cross-sectional	56.45 ± 13.60	7 (1–28) years	93.6%	healthy controls (*n* = 31)
**Sjögren Syndrome**							
McMillan et al., 2004 [23]	China	51	Monocentric cross-sectional	pSS: 50.1 ± 14.2, sSS: 43.3 ± 11.0	pSS: 6.7 ± 7.1, sSS: 4.8 ± 4.5 years	100%	healthy controls (*n* = 29)
Azuma et al., 2014 [24]	Japan	40	Monocentric cross-sectional	55.4 ± 13.2	5.6 ± 3.7 years	92.5%	non-Sjögren-syndrome * (*n* = 23)
Azuma et al., 2015 [25]	Japan	23	Observational study with 3 years follow-up	59.5 ± 12.7	5.5 ± 3.9 years	90.5%	healthy controls (*n* = 14)
Rusthen et al., 2017 [26]	Norway	31	Monocentric cross-sectional	52.0 ± 12.4	8.4 ± 8.2 years	100%	healthy controls (*n* = 33)
Nesvold et al., 2018 [27]	Norway	20	Monocentric cross-sectional	54.1 (34–70)	n/a	100%	no
Amaral et al., 2018 [28]	Portugal	86	Observational study with 2 weeks follow up	57.7 ± 13.1	n/a	98.8%	no
Fernandez-Martinez et al., 2019 [8]	Mexico	60	Monocentric cross-sectional	55.5 ± 8.1	7.6 ± 4 years	93.3%	healthy controls (*n* = 60)
daMata et al., 2019 [29]	Portugal	110	Randomized Clinical Trial	MA: 58.5 (55.3–61.8), CA 59.5 (56.5–62.6)	n/a	98.5%	no
**Behcet’s Disease**							
Mumcu et al., 2006 [30]	Turkey	94	Monocentric cross-sectional	33.6 ± 8.7	n/a	48.9%	healthy controls (*n* = 113)
Mumcu et al., 2007 [31]	Turkey	96	Monocentric cross-sectional	33.6 ± 8.7	n/a	50%	healthy controls (*n* = 117)
Mumcu et al., 2009 [32]	Turkey, UK	62	Multicentric cross-sectional	UK: 41.8 ± 11.5, Turkey: 41.5 ± 10.3	n/a	58.1%	no
Naito et al., 2014 [33]	Japan	675	Multicentric cross-sectional	55.5 ± 12.5	22 ± 12 years	48.3%	healthy controls (*n* = 1122)
**Systemic Lupus Erythematosus**							
Correa et al., 2018 [34]	Brazil	75	Monocentric cross-sectional	38.03 ± 9.80	n/a	90.7%	healthy controls (*n* = 75)
**Ankylosing Spondylitis**							
Schmalz et al., 2018 [12]	Germany	50	Monocentric cross-sectional	47.18 ± 15.67	10.92 ± 10.55	48%	healthy controls (*n* = 50)

OHRQoL: oral-health-related quality of life; UK: United Kingdom; pSS: primary Sjögren syndrome; sSS: secondary Sjögren syndrome; CSRG: Canadian Scleroderma Research Group; MA: malic acid group; CA: citric acid group; n/a: not applicable; *this control group included healthy individuals, alongside patients suffering from other rheumatic or chronic diseases.

**Table 2:** Applied assessments for OHRQoL and relevant results for the included studies.

Author, Year	Assessment of OHRQoL	OHRQoL Worse Than Control	Association and/or Correlation Between OHRQoL and General HRQoL	Association and/or Correlation Between OHRQoL and Oral Health	Association and/or Correlation Between OHRQoL and Rheumatic-Disease-Related Parameters
**Rheumatoid Arthritis**					
Blaizot et al., 2013 [9]	GOHAI: 47.4 ± 8.6	n/a	HAQ (dressing and grooming, eating, and walking)	M-T	no
Mühlberg et al., 2017 [13]	OHIP 14: 7.7 ± 9.6	yes (OHIP 14 control: 1.6 ± 2.1)	n/a	no	age
Chamani et al., 2017 [14]	OHIP 14: xerostomia: 10.97 ± 8.81, no xerostomia: 7.72 ± 7.10	n/a	n/a	DMF-T, xerostomia, denture wearing	disease duration, sex
Tristiu et al., 2018 [15]	Sc-GOHAI: 3.7 ± 2.5, OHIP 14: subjects reporting fairly or very often: 46.2%	GOHAI yes (Sc-GOHAI control: 1.36 ± 2.69), OHIP 14 no	GOHAI with RAPID 3	no	n/a
de Azevedo Branco et al., 2019 [11]	OHIP 49: 49.5 (9–132)	yes (OHIP 49 control: 23.0 (0–116))	n/a	DMF-T, frequency of tooth brushing	no
Nosratzehi et al., 2019 [16]	GOHAI: 37.46 ± 9.53	yes (GOHAI control: 53.21 ± 11.35)	n/a	n/a	age, number of involved joints, disease activity
Schmalz et al., 2020 [17]	OHIP 14: 5.4 ± 7.1 (2.5)	n/a	n/a	M-T	age, DAS-28, morning stiffness
**Systemic Sclerosis**					
Maddali Bongi et al., 2012 [10]	MHISS: 17.65 ± 5.20, dSSc: 37.5, lSSc: 41.4	n/a	no	mouth opening	no
Yuen et al., 2014 [19]	OHIP 49: baseline: 37.46 ± 36.92, 3 months: 28.46 ± 29.26, 6 months: 28.95 ± 35.47; OHIP 14: 9.97 ± 11.53, 7.41 ± 8.89, 7.44 ± 10.43	n/a	n/a	n/a	n/a
Baron et al., 2014 [20]	OHIP 49: 41.58 ± 32.53	yes (OHIP 49 control: 26.67 ± 25.15)	n/a	n/a	n/a
Baron et al., 2015 [21]	OHIP 49: 40.8 ± 32.4, dSSc: 37.5, lSSc: 41.4	n/a	SF-36 PSC and MSC	n/a	no
Parat et al., 2018 [22]	OHIP 49: 43.67 ± 21.06	yes (OHIP 49 control: 16.00 ± 19.60)	SHAQ	n/a	disease activity and severity, skin involvement, severity of general, skin, gastrointestinal, and joint/tendon involvement, and anti-topoisomerase I antibody
**Sjögren Syndrome**					
McMillan et al., 2004 [23]	OHIP 49: pSS: 39.4 ± 5.9, sSS: 37.0 ± 3.7	no (OHIP 49 control: 35.1 ± 5.2)	n/a	n/a	n/a
Azuma et al., 2014 [24]	OHIP 14: 11.3 ± 9.4	yes (OHIP 14 non-SS: 7.1 ± 7.6)	n/a	salivary flow	salivary flow, disease duration
Azuma et al., 2015 [25]	OHIP 14: baseline: 10.2 ± 8.8, follow-up: 12.6 ± 9.2	n/a	n/a	EGF in saliva	
Rusthen et al., 2017 [26]	OHIP 14: 16.2 ± 10.8	yes (OHIP 14 control: 2.7 ± 3.1)	n/a	dyseugesia, halitosis, gustatory score, salivary flow	age, salivary flow
Nesvold et al., 2018 [27]	OHIP 14: 14.0 (20.3) median (IQR)	n/a	n/a	no	n/a
Amaral et al., 2018 [28]	OHIP 14: baseline: 21.2 ± 11.7, follow-up: 21.0 ± 11.1	n/a	n/a	salivary flow	
Fernandez-Martinez et al., 2019 [8]	XeQoL 1.13 (0–3.8)	yes (XeQoL control: 0 (0–0.6))	SF-36, EPSSRI	salivary flow	
daMata et al., 2019 [29]	OHIP 14: baseline MA 21.5 ± 10, CA: 22 ± 12.2, follow up: MA 17.8 ± 10, CA 18.9 ± 11.4	n/a	n/a	no	no
**Behcet’s Disease**					
Mumcu et al., 2006 [30]	OHIP 14: 20.5 ± 14.0, OHRQoL questionnaire: 42.02 ± 11.4	yes (OHIP 14 control: 12.3 ± 15.5)	n/a	number of natural teeth	medication (colchicine vs. immunosuppressives) BD activity (active vs. inactive)
Mumcu et al., 2007 [31]	OHIP 14 active ulcers: 26.46 ± 13.14, inactive ulcers: 14.21 ± 12.98	yes (OHIP 14 control: 11.85 ± 12.14)	n/a	n/a	ulcer-related VAS and medication (colchicine vs. immunosuppressives)
Mumcu et al., 2009 [32]	OHIP 14: UK: 22.7 ± 14.4, T: 20.4 ± 14.3	n/a	n/a	extracted teeth	UK: healing time of ulcers, T: number of oral ulcers
Naito et al., 2014 [33]	GOHAI: 200 patients higher and 475 lower than Japanese norm (53.1)	yes*	n/a	n/a	active oral ulcers
**Systemic Lupus Erythematosus**					
Correa et al., 2018 [34]	OHIP 49: 43.0 (0) median (mode)	yes (OHIP 49 control: 22.00 (0))	n/a	denture wearing	SDI
**Ankylosing Spondylitis**					
Schmalz et al., 2018 [12]	OHIP 14: 6.2 (2; 0–10.75) mean (median; 25th–75th percentile)	yes (OHIP 14 control: 1.7 (0; 0 – 2.0))	n/a	no	BASDAI, BASFI, BAS-G, swollen joints, painful joints, morning stiffness, restriction of moving ability, problems with everyday things, physical pain and problems to care for himself

n/a: not applicable; OHRQoL: oral-health-related quality of life; OHIP: oral health impact profile; BD: Behcet’s disease; BASDAI: Bath Ankylosing Spondylitis Disease Activity Index; BASFI: Bath Ankylosing Spondylitis Functional Index; BAS-G: Bath Ankylosing Spondylitis Patient Global Score; HAQ: health assessment questionnaire; GOHAI: General Oral Health Assessment Index; RAPID3: Routine Assessment of Patient Index Data 3; SF-36: short-form 36 survey; EPSSRI: Emotional Problems Scales—Self Report Inventory; SDI: Systemic Lupus International Collaborating Clinics damage index; EGF: epidermal growth factor; DMF-T: decayed-, missing-, and filled-teeth index; M-T: number of missing teeth; XeQoL: Quality of Life in Xerostomia Questionnaire; dSSc: diffuse systemic sclerosis; lSSc: localized systemic sclerosis; pSS: primary Sjögren syndrome; sSS: secondary Sjögren syndrome; SDI: ; UK: United Kingdom; T: Turkey; MA: malic acid group; CA: citric acid group; MHISS: mouth handicap in systemic sclerosis; *no mean values for control available.

**Table 3:** Subscales of OHRQoL in included studies with OHIP questionnaire, presenting seven subscales.

OHIP 49								
Disease		Functional Limitation	Physical Pain	Psychosocial Discomfort	Physical Disability	Psycho-logical Disability	Social Disability	Handicap
**SSc** [20] mv ± sd	Disease	10.29 ± 7.39 *	10.39 ± 6.46 *	6.20 ± 5.23 *	6.68 ± 6.77 *	4.52 ± 5.06 *	1.33 ± 2.74 *	2.17 ± 3.84 *
	Control	6.08 ± 5.23	8.39 ± 5.90	4.29 ± 4.72	2.84 ± 4.33	3.17 ± 4.20	0.68 ± 1.89	1.22 ± 3.01
**SS** [23] mv ± sd	Disease pSS/sSS	11.9 ± 1.3/11.6 ± 1.0	8.9 ± 1.4/9.0 ± 0.8	4.4 ± 0.9/5.4 ± 0.9	7.0 ± 1.2/4.8 ± 5.4	3.3 ± 0.9/3.0 ± 0.7	1.0 ± 0.4/0.9 ± 0.3	3.0 ± 0.8/2.4 ± 0.4
	Control	10.2 ± 1.2	8.8 ± 1.1	4.9 ± 0.8	4.7 ± 1.0	3.3 ± 0.8	1.0 ± 0.4	2.2 ± 0.6
**RA** with/without xerostomia [14] mv ± sd	Disease with/without xerostomia	1.34 ± 1.47/0.95 ± 1.25	2.43 ± 2.25/1.58 ± 1.52	1.60 ± 1.63/1.50 ± 1.65	1.55 ± 1.93/0.79 ± 1.31	1.69 ± 2.04/1.47 ± 1.74	1.05 ± 1.67/0.63 ± 1.23	1.27 ± 2.17/0.76 ± 1.77
	Control	-	-	-	-	-	-	-
**RA** [11] median (range)	Disease	15.0 (3–30) *	10.5 (2–28)	6.5 (0–20)	6.0 (0–25) *	3.0 (0–23)	0 (0–16)	0 (0–12)
	Control	10.0 (0–28)	9.5 (0–26)	5.5 (0–20)	1.0 (0–28)	0.5 (0–17)	0 (0–16)	0 (0–12)
**SLE** [34] median (mode)	Disease	14.0 (0)	12.0 (0)	8.0 (0)	4.0 (0) *	3.0 (0)	0 (0)	0 (0)
	Control	9.0 (0)	9.0 (0)	2.0 (0)	1.0 (0)	0 (0)	0 (0)	0 (0)
**OHIP 14**								
**Disease**		**Functional Limitation**	**Physical Pain**	**Psychosocial Discomfort**	**Physical Disability**	**Psycho-** **logical Disability**	**Social Disability**	**Handicap**
**SS** [28] mv ± sd	Disease	2.9 ± 2.1	3.7 ± 2.2	4.3 ± 2.6	2.5 ± 2.3	2.8 ± 2.1	2.3 ± 2.0	2.7 ± 2.2
	Control	-	-	-	-	-	-	-
**SS** [29] mv ± sd	Disease MA/CA	3.1 ± 1.8/2.9 ± 2	3.7 ± 1.7/3.8 ± 2.1^b^	4.5 ± 2.2/4.4 ± 2.7	2.7 ± 2.0/2.7 ± 2.4	3.0 ± 1.9/2.9 ± 2	2.2 ± 2/2.6 ± 2.1	2.4 ± 1.9/2.8 ± 2.2
	Control	-	-	-	-	-	-	-

* Significantly worse than healthy control group; OHIP: oral health impact profile; SSc: systemic sclerosis; pSS: primary Sjögren syndrome; sSS: secondary Sögren syndrome; RA: rheumatoid arthritis; BD: Behcet’s disease; SLE: systemic lupus erythematosus; MA: malic acid group; CA: citric acid group; mv: mean value; sd: standard deviation.

**Table 4:** Findings of studies which applied alternative subscales of the OHIP 14 questionnaire.

OHIP 14					
Disease		Oral Function	Psychosocial Impact	Oral Pain	Orofacial Appearance
**RA** [17] mv ± sd	Disease	2.0 ± 2.8	2.4 ± 4.0	0.7 ± 1.1	0.4 ± 0.8
	Control	-	-	-	-
**Disease**		**Physical Symptoms**		**Psychosocial Symptoms**	**Psychological Symptoms**
**BD** active/inactive ulcers [31] mv ± sd	Disease	9.14 ± 5.45/5.01 ± 4.97 *		8.48 ± 5.56/4.15 ± 4.87 *	8.78 ± 4.44/5.04 ± 4.10 *
	Control	3.37 ± 4.49		3.86 ± 4.72	4.19 ± 3.68
**BD** UK/Turkey [32] mv ± sd	Disease	8.7 ± 5.2/7.01 ± 5.7		8.3 ± 5.5/6.6 ± 5.5	5.7 ± 4.3/7.1 ± 4.9
	Control	-		-	-

* Significantly worse than healthy control group; OHIP: Oral Health Impact Profile; RA: rheumatoid arthritis; BD: Behcet’s disease; mv: mean value; sd: standard deviation.

**Table 5:** Validity of the applied questionnaires for assessment of OHRQoL in patients with rheumatoid diseases.

Disease	Questionnaire	ICC	Crohnbach’s α	Further Results
**SSc** [19]	OHIP 49	0.84 (t0–3 mo), 0.69 (3 mo–6 mo)	n/a	SEM 12.05 (t0–3 mo), 18.34 (3 mo–6 mo)
**SSc** [19]	OHIP 14	0.82 (t0–3 mo), 0.61 (3 mo–6 mo)	n/a	SEM 4.07 (t0–3 mo), 6.09 (3 mo–6 mo)
**SSc** [10]	MHISS	0.93	0.99	n/a
**SS** [28]	OHIP 14	0.94	0.89	n/a
**SS** [29]	OHIP 14	n/a	n/a	SEM: 3.3 or 4.0 depending on intervention
**BD** [30]	OHIP 14	n/a	0.92	Test–retest correlation 0.64–0.79
**BD** [30]	OHRQoL questionnaire	n/a	0.93	Test–retest correlation 0.67–0.99
**BD** [31]	OHIP 14	n/a	0.91 (active), 0.93 (inactive)	n/a
**BD** [32]	OHIP 14	n/a	0.95 (UK) and 0.93 (Turkey)	n/a

OHIP: Oral Health Impact Profile; OHRQoL: oral-health-related quality of life; ICC: intraclass correlation; SEM: standard eroor of measurement; UK: United Kingdom; SSc: systemic sclerosis; SS: Sjörgen syndrome; BD: Behcet’s disease; mo: month.

## Supplementary Materials

The following are available online at https://www.mdpi.com/2077-0383/9/4/1172/s1, Table S1: Excluded studies during the evaluation process with reasons for exclusion, Table S2. Oral health parameters that were examined related to OHRQoL in the included studies.

## Abbreviations

AS	Ankylosing spondylitis
BASDAI	Bath Ankylosing Spondylitis Disease Activity Index
BASFI	Bath Ankylosing Spondylitis Functional Index
BAS-G	Bath Ankylosing Spondylitis Patient Global Score
BD	Behcet disease
CA	citric acid group
CAL	clinical attachment loss
CSRG	Canadian Scleroderma Research Group
DAS 28	disease activity score 28
DMF-T	decayed-, missing-, and filled-teeth index
dSSc	diffuse systemic sclerosis
D-T	number of decayed teeth
EPSSRI	Emotional Problems Scales-Self Report Inventory
EGF	epidermal growth factor
GOHAI	general oral health assessment index
HAQ	health assessment questionnaire
HRQoL	health-related quality of life
ICC	intraclass correlation
lSSc	localized systemic sclerosis
MA	malic acid group
MHISS	mouth handicap in systemic sclerosis
M-T	number of missing teeth
n/a	not applicable
OHIP	oral health impact profile
OHRQoL	oral-health-related quality of life
PRISMA	Preferred Reporting Items for Systematic Reviews and Meta-Analyses
pSS	primary Sjögren syndrome
RA	rheumatoid arthritis
RAPID3	Routine Assessment of Patient Index Data 3
SDI	SLICC damage index
SEM	standard error of measurement
SF-36	short form 36 survey
SLE	systemic lupus erythematosus
SS	Sjögren Syndrome
sSS	secondary Sjögren Syndrome
SSc	Systemic sclerosis
T	Turkey
UK	United Kingdom
XeQoL	quality of life questionnaire in xerostomy
UK	United Kingdom
XeQoL	quality of life questionnaire in xerostomy
PICO	patient intervention control outcome
