# Supplementary material for: Oral-Health-Related Quality of Life in Adult Patients with Rheumatic Diseases—A Systematic Review
Source: J Clin Med. 2020 Apr 19;9(4):1172. doi: 10.3390/jcm9041172 (PMC7231140; doi:10.3390/jcm9041172)
Supplement: Supplementary file 1 [file jcm-09-01172-s001.pdf]

Journal of Clinical Medicine

MDPI

Table S1. Excluded studies during the evaluation process with reasons for exclusion.

Author and year	Issue of examination	Reason for exclusion
John et al. 2007	OHRQoL of patients with temporomandibular disorders	No examination of patients with rheumatic diseases
Rener-Sitar et al. 2008	OHRQoL of patients with craniomandibular disorders	No examination of patients with rheumatic diseases
Larsson 2010	Evaluation of OHRQoL assessment tools in Swedish polulation	No examination of patients with rheumatic diseases
Rener-Sitar et al. 2013	Impact of temporomandibular disorders on OHRQoL	No examination of patients with rheumatic diseases
Su et al. 2014	Influence of hyaluronic acid injection on OHRQoL of patients with temporomandibular joint osteoarthritis	No examination of patients with rheumatic diseases (osteoarthritis restricted to the temporomandibular joint)
Ahola et al. 2015	OHRQoL of patients with rheumatic diseases	No verified diagnosis of rheumatic diseases (survey via mail)
Santos et al. 2015	OHRQoL of patients with idiopathic juvenile arthritis	Age < 18 years
Su et al. 2016	OHRQoL of patients with temporomandibular joint osteoarthritis	No examination of patients with rheumatic diseases (osteoarthritis restricted to the temporomandibular joint)
Su et al. 2018	OHRQoL of patients with temporomandibular joint osteoarthritis	No examination of patients with rheumatic diseases (osteoarthritis restricted to the temporomandibular joint)
Rahimi et al. 2018	OHRQoL of patients with idiopathic juvenile arthritis	Age < 18 years
Isola et al. 2019	OHRQoL of patients with idiopathic juvenile arthritis	Age < 18 years
Su et al. 2019	OHRQoL of patients with temporomandibular joint osteoarthritis	No examination of patients with rheumatic diseases (osteoarthritis restricted to the temporomandibular joint)
Bucci et al. 2019	OHRQoL of patients with idiopathic juvenile arthritis	Age < 18 years

OHRQoL: oral health-related quality of life

Supplementary table S1 References:

1. John, M.T., Reissmann, D.R., Schierz, O., Wassell, R.W. Oral health-related quality of life in patients with temporomandibular disorders. J Orofac Pain. 2007, 21, 46-54.
2. Rener-Sitar, K., Celebić, A., Stipetić, J., Marion, L., Petricević, N., Zaletel-Kragelj, L. Oral health related quality of life in Slovenian patients with craniomandibular disorders. Coll Antropol. 2008, 32, 513-517.
3. Larsson, P. Methodological studies of orofacial aesthetics, orofacial function and oral health-related quality of life. Swed Dent J Suppl. 2010, 11-98.
4. Rener-Sitar, K., Celebić, A., Mehulić, K., Petricević, N. Factors related to oral health related quality of life in TMD patients. Coll Antropol. 2013, 37, 407-413.
5. Su, N., Yang, X., Liu, Y., Huang, Y., Shi, Z. Evaluation of arthrocentesis with hyaluronic acid injection plus oral glucosamine hydrochloride for temporomandibular joint osteoarthritis in oral-health-related quality of life. J Craniomaxillofac Surg. 2014, 42(6), 846-851.
6. Ahola, K., Saarinen, A., Kuuliala, A., Leirisalo-Repo, M., Murtomaa, H., Meurman, J.H. Impact of rheumatic diseases on oral health and quality of life. Oral Dis. 2015, 21, 342-348.

7. Santos, D., Silva, C., Silva, M. Oral health and quality of life of children and adolescents with juvenile idiopathic arthritis according to their caregivers' perceptions. Spec Care Dentist. 2015, 35, 272-278.
8. Su, N., Liu, Y., Yang, X., Shen, J., Wang, H. Correlation between oral health-related quality of life and clinical dysfunction index in patients with temporomandibular joint osteoarthritis. J Oral Sci. 2016, 58, 483-490.
9. Su, N., Liu, Y., Yang, X., Shen, J., Wang, H. Association of malocclusion, self-reported bruxism and chewing-side preference with oral health-related quality of life in patients with temporomandibular joint osteoarthritis. Int Dent J. 2018, 68, 97-104.
10. Rahimi, H., Twilt, M., Herlin, T., Spiegel, L., Pedersen, T.K., Küseler, A., Stoustrup, P. Orofacial symptoms and oral health-related quality of life in juvenile idiopathic arthritis: a two-year prospective observational study. Pediatr Rheumatol Online J. 2018, 16, 47.
11. Isola, G., Perillo, L., Migliorati, M., Matarese, M., Dalessandri, D., Grassia, V., Alibrandi, A., Matarese, G. The impact of temporomandibular joint arthritis on functional disability and global health in patients with juvenile idiopathic arthritis. Eur J Orthod. 2019, 41, 117-124.
12. Su, N., Wang, H., van Wijk, A.J., Visscher, C.M., Lobbezoo, F., Shi, Z., van der Heijden, G.J.M.G. Prediction Models for Oral Health-Related Quality of Life in Patients with Temporomandibular Joint Osteoarthritis 1 and 6 Months After Arthrocentesis with Hyaluronic Acid Injections. J Oral Facial Pain Headache. 2019, 33, 54–66.
13. Bucci, R., Rongo, R., Amato, A., Martina, S., D'Antò, V., Valletta, R. The Psychological Impact of Dental Aesthetics in Patients with Juvenile Idiopathic Arthritis Compared with Healthy Peers: A Cross-Sectional Study. Dent J (Basel). 2019, 7(4).

Table S2. Oral health parameters that were examined related to OHRQoL in the included studies.

Author, year	Dental paramers	Periodontal parameters	Other oral health parameters
**Rheumatoid arthritis**			
Blaizot et al. 2013 [9]	number of teeth, need for conservative dental treatment, need for prosthetic treatment	need for periodontal treatment	no
Mühlberg et al. 2017 [13]	DMF-T	periodontal disease severity	no
Chamani et al. 2017 [14]	DMF-T, denture wearing	no	xerostomia
Tristiu et al. 2018 [15]	tooth loss, denture wearing	periodontitis severity	no
de Azevedo Branco et al. 2019 [11]	DMF-T, M-T, denture wearing	presence of periodontitis	stimulated and unstimulated salivary flow
Nosratzehi et al. 2019 [16]	no	no	no
Schmalz et al. 2020 [17]	dental treatment need, M-T	no	no
**Systemic sclerosis**			
Maddali Bongi et al. 2012 [10]	no	no	mouth opening
Yuen et al. 2014 [19]	no	no	no

Baron et al. 2014 [20]	D-T, F-T, M-T	number of teeth with probing depth >3mm or CAL ≥5.5	interincisal distance, salivary flow
Baron et al. 2015 [21]	no	no	no
Parat et al. 2018 [22]	no	no	no
**Sjögren Syndrome**			
McMillan et al. 2004 [23]	no	no	no
Azuma et al. 2014 [24]	no	no	salivary flow, salivary level of epidermal growth factor
Azuma et al. 2015 [25]	no	no	salivary level of epidermal growth factor
Rusthen et al. 2017 [26]	no	no	stimulated and unstimulated salivary flow
Nesvold et al. 2018 [27]	no	no	stimulated and unstimulated salivary flow
Amaral et al. 2018 [28]	no	no	stimulated and unstimulated salivary flow
Fernandez-Martinez et al. 2019 [8]	no	no	unstimulated salivary flow
daMata et al. 2019 [29]	denture wearing	periodontal status	salivary output
**Behcet´s disease**			
Mumcu et al. 2006 [30]	number of teeth	no	oral apthous ulcers
Mumcu et al. 2007 [31]	extracted teeth	no	oral apthous ulcers
Mumcu et al. 2009 [32]	carious teeth, extracted teeth, filled teeth, number of teeth, Plaque index	gingival index, Sulcus bleeding index, clinical attachment level	oral apthous ulcers
Naito et al. 2014 [33]	no	no	oral apthous ulcers
**Systemic lupus erythematosus**			
Correa et al. 2018 [34]	DMF-T, D-T, F-T, M-T, denture wearing	presence of periodontitis	stimulated and unstimulated salivary flow
**Ankylosing Spondylitis**			
Schmalz et al. 2018 [12]	DMF-T, D-T, M-T	periodontal disease severity	no

DMF-T: decyed-, missing- and filled teeth index, D-T: number of decaed teeth, F-T: number of filled teeth, M-T: number of missing teeth, CAL: clinical attachment loss.
